# *In silico *genomic analyses reveal three distinct lineages of *Escherichia coli *O157:H7, one of which is associated with hyper-virulence

**DOI:** 10.1186/1471-2164-10-287

**Published:** 2009-06-29

**Authors:** Chad R Laing, Cody Buchanan, Eduardo N Taboada, Yongxiang Zhang, Mohamed A Karmali, James E Thomas, Victor PJ Gannon

**Affiliations:** 1 Laboratory for Foodborne Zoonoses, Public Health Agency of Canada, Lethbridge, AB, Canada; 2 Laboratory for Foodborne Zoonoses, Public Health Agency of Canada, Guelph, ON, Canada; 3 Faculty of Biological Sciences, University of Lethbridge, Lethbridge, AB, Canada

## Abstract

**Background:**

Many approaches have been used to study the evolution, population structure and genetic diversity of *Escherichia coli *O157:H7; however, observations made with different genotyping systems are not easily relatable to each other. Three genetic lineages of *E. coli *O157:H7 designated I, II and I/II have been identified using octamer-based genome scanning and microarray comparative genomic hybridization (mCGH). Each lineage contains significant phenotypic differences, with lineage I strains being the most commonly associated with human infections. Similarly, a clade of hyper-virulent O157:H7 strains implicated in the 2006 spinach and lettuce outbreaks has been defined using single-nucleotide polymorphism (SNP) typing. In this study an *in silico *comparison of six different genotyping approaches was performed on 19 *E. coli *genome sequences from 17 O157:H7 strains and single O145:NM and K12 MG1655 strains to provide an overall picture of diversity of the *E. coli *O157:H7 population, and to compare genotyping methods for O157:H7 strains.

**Results:**

*In silico *determination of lineage, Shiga-toxin bacteriophage integration site, comparative genomic fingerprint, mCGH profile, novel region distribution profile, SNP type and multi-locus variable number tandem repeat analysis type was performed and a supernetwork based on the combination of these methods was produced. This supernetwork showed three distinct clusters of strains that were O157:H7 lineage-specific, with the SNP-based hyper-virulent clade 8 synonymous with O157:H7 lineage I/II. Lineage I/II/clade 8 strains clustered closest on the supernetwork to *E. coli *K12 and *E. coli *O55:H7, O145:NM and sorbitol-fermenting O157 strains.

**Conclusion:**

The results of this study highlight the similarities in relationships derived from multi-locus genome sampling methods and suggest a "common genotyping language" may be devised for population genetics and epidemiological studies. Future genotyping methods should provide data that can be stored centrally and accessed locally in an easily transferable, informative and extensible format based on comparative genomic analyses.

## Background

*Escherichia coli *O157:H7 is the most commonly implicated serotype of Shiga-toxin producing *E. coli *(STEC) or enterohemorrhagic *E. coli *(EHEC) associated with hemorrhagic colitis and the hemolytic uremic syndrome (HUS) [1,2]. It is recognized world-wide as an important cause of both sporadic cases and outbreaks of food- and waterborne disease. Genomic diversity within populations of this pathogen is extensive and genome comparisons have revealed many DNA insertion/deletion and recombination events which are thought to be driven primarily through bacteriophage-mediated and other mechanisms of lateral gene transfer [3-5].

Three genetic lineages of *E. coli* O157:H7 have been described using octamer-based genome scanning (OBGS) and microarray-based comparative genomic hybridization (mCGH). Lineage I strains are commonly isolated from both cattle and clinically ill humans, lineage II strains are isolated primarily from cattle and intermediate lineage I/II strains are less well characterized with respect to phenotype and host distribution [6-8]. Additional studies on the evolution, population structure and genetic diversity of *Escherichia coli *O157:H7 have been carried out using a number of different genotyping approaches, each of which is based on targeting polymorphisms in a particular locus or set of loci. For example, the presence of Shiga-toxin (Stx) containing bacteriophage integration sites has been used to describe sixteen *E. coli *O157:H7 genotypes (with the majority associated with strains of bovine origin) [9]. Multi-locus variable number tandem repeat analysis (MLVA), which targets the number of repeats at nine genetic loci in the method proposed for PulseNet, has recently been used for epidemiological typing of O157:H7 strains [10]. mCGH, which examines the presence or absence of every gene in the genome of a reference strain or strains, has been used to elucidate the step-wise emergence of O157:H7 strains from an O55:H7-like ancestor [11] and by our laboratory to first characterize lineage I/II strains [7]. Single-nucleotide polymorphism (SNP) typing examines the single nucleotide changes throughout the *E. coli *O157:H7 genome; SNPs in 96 loci have been used to delineate 39 genotypes in over 500 strains, which have been partitioned into nine evolutionary clades [12]. One of these clades (clade 8) was found to contain putative hyper-virulent strains, including those implicated in the 2006 spinach- and lettuce-associated outbreaks in the United States. Comparative genomic fingerprinting (CGF), which examines the presence or absence of the most common variable loci within the genome, has recently been used with 23 loci to analyze 79 O157:H7 strains, and to group them into lineages and epidemiologically and phenotypically related clusters [13].

The various methods have proved useful on their own but in most cases the strain groupings from one method are not easily relatable to another, and none of these methods has yet achieved the "gold standard" status to be used as a common genotyping method. Because each of these genotyping approaches can provide important information on isolates with different pathogenic and virulence characteristics, the need to replicate each of these methods using a common group of reference strains has arisen. However, present restrictions on international strain exchange make the acquisition of certain reference strains nearly impossible for researchers outside the country of origin of the strains. While the stepwise emergence of *E. coli *O157:H7 from an O55:H7-like serotype has been advanced [11,14], the relationship among *E. coli *O157:H7 lineages and clades incorporating data from standard and more advanced genotyping methods has not been evaluated. Thus, discoveries such as the recently described hyper-virulent clade of *E. coli *O157:H7 strains [12] are framed only in the context of the individual study.

Recently, three complete *E. coli *O157:H7 strain sequences and 11 whole genome shotgun sequences have become available in Genbank. With the additional sequencing of one O157:H7 and one O145:NM strain by our group, the availability of multiple whole-genome sequences has allowed us to apply several molecular typing schemes *in silico *to a group of *E. coli *O157:H7 and related strains. We have applied six molecular typing methods shown to differentiate among *E. coli *O157:H7 strains to a panel of nineteen genome-sequenced strains: Stx-bacteriophage insertion site typing [9], CGF [13], mCGH [7,11], SNP genotyping [12], genomic *in silico *subtractive hybridization (GISSH) (Laing *et al*. in preparation) and MLVA[10].

In this study we provide a view of the relationships among *E. coli *O157:H7 strains based on a supernetwork representation of these six separate molecular typing methods. We also show how hyper-virulent clade 8 *E. coli *O157:H7 strains fit into this scheme and suggest the use of genotyping approaches that are relatable as part of a "common genotyping language" until whole genome sequencing becomes routine in bacterial strain genotyping.

## Results

### Lineage typing

The *is-*PCR LSPA6 typing of the *E. coli *O157:H7 strains in Table 1 designated strains EC4501 and TW14588 as lineage I, eleven of the 14 GenBank strains and the in-house strain EC71074 as lineage I/II, and strain EC869 as lineage II.

**Table 1:** The 19 *E. coli *strains analyzed *in silico *in this study.

Strain	Serotype	Sequence source	LSPA6 lineage	SNP clade	Stx-phage insertion site genotype
EC4024	O157:H7	NZ_ABJT00000000	I/II	8	1
EC4042	O157:H7	NZ_ABHM00000000	I/II	8	1
EC4045	O157:H7	NZ_ABHL00000000	I/II	8	1
EC4076	O157:H7	NZ_ABHQ00000000	I/II	8	1
EC4113	O157:H7	NZ_ABHP00000000	I/II	8	1
EC4115	O157:H7	NZ_ABHN00000000	I/II	8	1
EC4196	O157:H7	NZ_ABHO00000000	I/II	8	1
EC4206	O157:H7	NZ_ABHK00000000	I/II	8	1
EC4401	O157:H7	NZ_ABHR00000000	I/II	8	1
EC4486	O157:H7	NZ_ABHS00000000	I/II	8	1
EC508	O157:H7	NZ_ABHW00000000	I/II	8	1
EC71074	O157:H7	Public Health Agency of Canada	I/II	8	1
EC869	O157:H7	NZ_ABHU00000000	II	uncertain	6
EC4501	O157:H7	NZ_ABHT00000000	I	2	3
TW14588	O157:H7	NZ_ABKY00000000	I	2	3
EDL933	O157:H7	NC_002655.2	I	3	3
Sakai	O157:H7	NC_002695.1	I	1	3
EC33264	O145:NM	Public Health Agency of Canada	NA	NA	NA
MG1655	K12	NC_000913.2	NA	NA	NA

### Stx-phage insertion site typing

Besser et al. [9] found greater diversity within O157:H7 strains isolated from cattle than those from human clinical illness, but the relationship between their typing scheme and lineage groupings was not explored. In Table 1 it is evident from *in silico *analysis that all genotypes based on the Stx-phage integration site typing can be related to mCGH lineage.

All lineage I strains were of genotype 3 in that they possessed both *stx*_1 _and *stx*_2 _and both bacteriophage integration sites *yehV *and *wrbA *were occupied. Additionally, they all possessed the "K" form of the N135K polymorphism in the FimH mannose binding pocket, lacked *stx*_2c_, had the "T" form of the T238A polymorphism in *tir *and were positive for the *Q*-*stx*_2 _junction of phage 933W.

All lineage I/II strains were of genotype 1 in that they contained *stx*_2 _but not *stx*_1 _and contained an occupied *yehV *and an intact *wrbA *bacteriophage integration site. Additionally, they all possessed the "N" form of the N135K polymorphism in the FimH mannose binding pocket and the "T" form of the T238A polymorphism in *tir*. However, the *Q*-*stx*_2 _junction of phage 933W and *stx*_2c _were variably present among the lineage I/II strains.

Lineage II strain EC869 was of genotype 6. It contained both *stx*_1 _and *stx*_2 _had the variant-R form of the *yehV *bacteriophage right junction and an intact *wrbA *integration site. EC869 possessed *stx*_2c_, the "A" form of the T238A polymorphism in *tir*, the "N" form of the N135K polymorphism in the FimH mannose binding pocket and was negative for the *Q*-*stx*_2 _junction of phage 933W. The raw data for this and all other *in silico *typing methods are provided [see Additional file 1].

### SNP genotyping

The clade of each strain based on SNP typing was determined as described by Riordan et al. [15] where SNPs in the four genes ECs2357, ECs2521, ECs3881 and ECs4130 allow the delineation of the four most common clades of *E. coli *O157:H7. Our analysis typed EDL933 as clade 3 (SNP profile in the gene order given above as CGCT), Sakai as clade 1 (SNP profile CTTT), TW14588 and EC4501 as clade 2 (SNP profile CGTT), EC869 as one of clades 4–7 (SNP profile CGCC) and the remaining O157:H7 strains as members of the hyper-virulent clade 8 (SNP profile AGCC) (Table 1).

### GISSH-based novel region distribution

**Figure 1:**
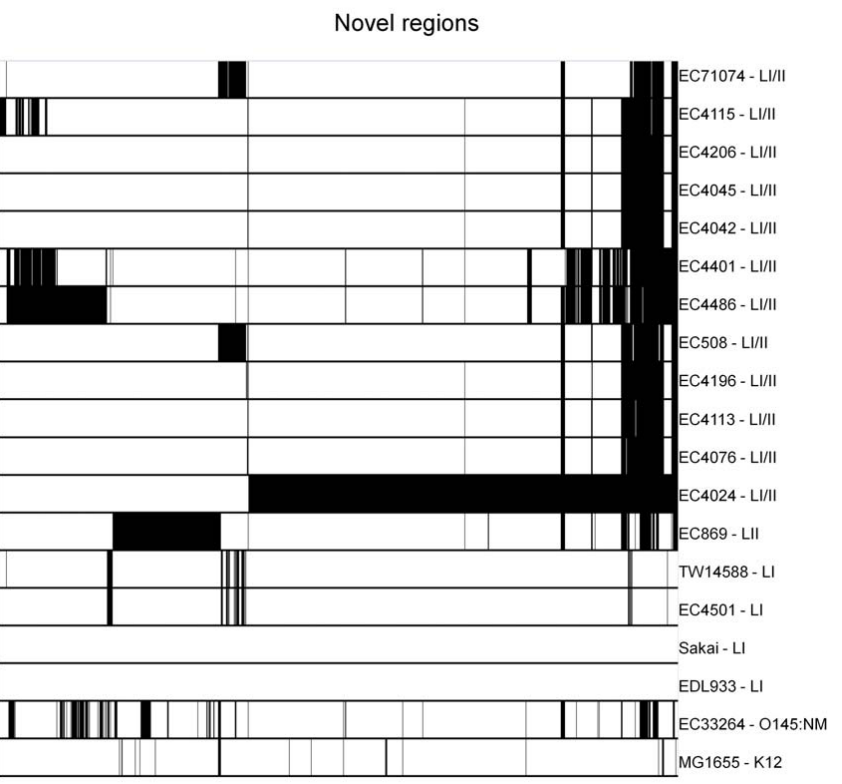
**Novel regions distribution among the *E. coli *strains in Table 1**. The distribution of 1456 regions ~500 bp in size among 17 *E. coli *O157:H7 strains and the O145:NM strain EC33264 and K12 strain MG1655. Regions are not necessarily contiguous and are defined as novel based on less than 80% sequence identity to the genome of either  EDL933 or Sakai. Black indicates the presence of a region and white indicates the absence of a region.

The microarray used in the study by Zhang et al. [7] was based on lineage I strains EDL933 and Sakai and the K12 strain MG1655, so it is not surprising that the lineage I strains were found to contain more of the lineage I markers than strains from the other lineages. To account for novel genomic regions present in other O157:H7 strains, but not found in EDL933 or Sakai, an *in silico *subtractive hybridization was performed on every O157:H7 sequence in Table 1 against EDL933 and Sakai (Laing et al., in preparation). The study found 417 separate regions comprising 1456 segments of approximately 500 bp in length (~0.8 Mbp of novel DNA sequence). The distribution of these segments *in silico *is shown in Figure 1 and highlights the fact that lineage I strains possess genomic regions not represented in the original microarray probe set, as well as the fact that there are other lineage I/II and lineage II specific genomic regions.

### Combined analysis

The maximum parsimony trees from each method shared a number of commonalities [see Additional file 2]. All three lineages grouped distinctly with the following methods: CGF, SNP genotyping, mCGH and GISSH-based novel region typing. Stx-phage insertion site typing distinguished lineage I strains as a separate cluster but lineage II strain EC869 did not form a distinct cluster from the lineage I/II strains. Similarly, MLVA put lineage II strain EC869 on its own branch, whereas the lineage I/II and lineage I strains did not group separately with this method. All of the genotyping methods except mCGH identified lineage I/II strains EC508 and EC71074 as a separate cluster, which was placed into a group close to the lineage I strains in the CGF and Stx-phage insertion site based maximum parsimony trees and was placed between lineage I strain Sakai and the other three lineage I strains in the maximum parsimony tree based on MLVA data.

**Figure 2:**
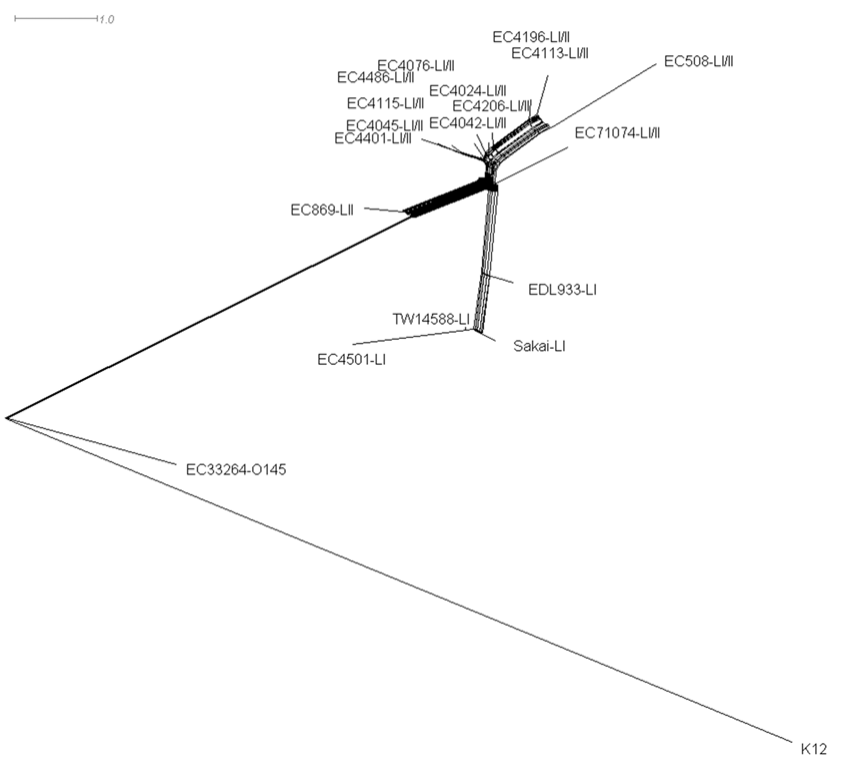
**Supernetwork constructed from *in silico *data of six typing methods**. The supernetwork created from the combination of each maximum parsimony tree from the following typing methods: Stx-phage insertion site typing, MLVA, CGF, SNP genotyping, mCGH, and GISSH-based novel region distribution typing. Maximum parsimony trees were combined using the un-weighted mean distance and Z-closure with 1000 iterations; the resulting supernetwork was displayed using the equal angle method.

In order to obtain a more complete understanding of the relationships among *E. coli *O157:H7 strains, the maximum parsimony trees derived using *in silico *data from the six separate molecular typing methods included in this study were combined to form a supernetwork. We accomplished this by combing the six maximum parsimony trees generated using PAUP* instead of concatenating the data into a single matrix and generating a single most parsimonious tree. This ensured a method with many data points such as mCGH did not outweigh a method like MLVA that contains few data points. The supernetwork based on the six trees given equal weighting (Figure 2) shows competing signals, rather than an inferred absolute tree [16]. As can be seen, the 19 strains were distributed into four main groups. One contained strains K12 MG1655 and O145:NM EC33264 and another the single lineage II strain EC869. The remaining two groups of the supernetwork were lineage-specific, comprising one cluster of lineage I strains and another of lineage I/II strains. The distribution of the four lineage I strains was consistent with the clade breakdown of Manning et al. [12], as the clade 2 strains TW14588 and EC4501 were more closely related to each other than clade 3 strain EDL933 or clade 1 strain Sakai.

**Figure 3:**
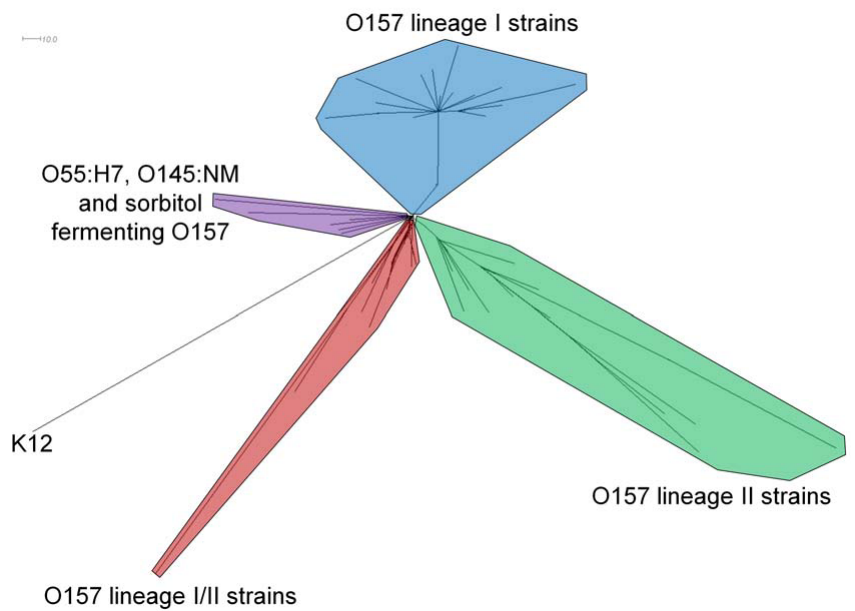
**Supernetwork constructed from *in silico *data of six typing methods and experimental data from two methods**. The supernetwork created from the combination of each maximum parsimony tree from the following typing methods: Stx-phage insertion site typing, MLVA, CGF, SNP genotyping, mCGH, and GISSH-based novel region distribution typing. Both mCGH and CGF datasets included the *in silico *data from the strains in Table 1 in addition to experimental data from the remaining strains in Table 2. Maximum parsimony trees were combined using the un-weighted mean distance and Z-closure with 1000 iterations; the resulting supernetwork was displayed using the equal angle method.

Because only a relatively small number of strains have been sequenced, we repeated the analysis including experimental microarray [7,11] and CGF data [13] for which lineage information was available to provide a larger context for our *in silico *analysis. This added 83 strains to the 19 strain *in silico *dataset and included data for three *E. coli *O55:H7 strains and two sorbitol-fermenting *E. coli *O157:H-strains (Table 2), allowing us to frame the resulting supernetwork within the context of the proposed stepwise emergence of *E. coli *O157:H7 [11]. The supernetwork in Figure 3 again showed four main groups. All lineage I and II strains formed discrete branches in the supernetwork, while the lineage I/II strains formed a cluster that was closest to *E. coli *K12 and strains of O55:H7, O145:NM and sorbitol-fermenting O157:H-.

**Table 2:** The 102 *E. coli *O157:H7 strains used in the construction of the supernetwork in Figure 3.

Strain	Lineage	Strain	Lineage	Strain	Lineage
		
AA10002	I	H2727	I	EC4113	I/II
AA10021	I	H2731	I	EC4115	I/II
APF593	I	H432	I	EC4196	I/II
2328	I	H435	I	EC4206	I/II
23339	I	H4420	I	EC4401	I/II
58212	I	H451	I	EC4486	I/II
63154	I	H453	I	EC508	I/II
70490	I	H454	I	EC71074	I/II
813601	I	H568	I	R1388	I/II
93111	I	H571	I	Zap0046	I/II
97701	I	H572	I	AA6192	II
EC4501	I	H573	I	AA9952	II
EC980120	I	H574	I	E12491	II
EC980121	I	LN6374	I	EC19920026	II
EC980122	I	LRH6	I	EC869	II
EC980125	I	LRH73	I	EC970520	II
EDL933	I	LS110	I	F1081	II
F1082	I	LS236	I	F12	II
F1095	I	M01MD3265	I	F1305	II
F1103	I	OK1	I	FRIK1985	II
F1299	I	R1195	I	FRIK1990	II
F2	I	S23021	I	FRIK1999	II
F30	I	S2628	I	FRIK2001	II
F5	I	S3722	I	FRIK920	II
F732	I	Sakai	I	LRH13	II
F744	I	TS97	I	LS68	II
H2160	I	TW14588	I	R1797	II
H2161	I	09601Fe046.1	I/II	493/89	O157:H-
H2163	I	32511	I/II	CB2755	O157:H-
H2164	I	59243	I/II	Dec5d	O55:H7
H2176	I	EC4024	I/II	TB182A	O55:H7
H2704	I	EC4042	I/II	5905	O55:H7
H2718	I	EC4045	I/II	EC33264	O145:NM
H2723	I	EC4076	I/II	MG1655	K12

## Discussion

The acquisition and loss of genetic elements in *E. coli *O157:H7 is thought to affect the virulence of this pathogen in humans [17-19]. While attributes like the Stxs [20-22] and the locus of enterocyte attachment and effacement (LEE) [23] are well known, other less well characterized elements such as non-LEE effectors (NLE)s [24] may contribute to the spectrum of virulence that has been captured for STEC within the seropathotype classification [25].

This genomic diversity can largely be attributed to bacteriophages [4,26] and other mobile elements [27] that cause DNA segment insertion and deletion events, rather than single-nucleotide changes [28]. The best characterization of diversity within *E. coli *O157:H7 must therefore take both single-nucleotide changes and large region turnover events into consideration. Whole genome sequencing is the best method for such analysis, but until the number of completely sequenced strains increases and whole genome sequencing becomes both routine and cost-effective, an estimation of whole genomic change based on sampling of polymorphisms or variability at a number of specific loci will have to suffice. A number of molecular genotyping methods have recently emerged that target different regions and types of variation, so the combination of data from these methods can offer a picture that is greater than the sum of their individual views. It can be argued that lateral gene transfer obscures the relationship among bacterial strains; however, once mobile elements are acquired and if they are stably maintained, they can be especially valuable in assessing strain relationships. We therefore advocate the use of genotyping methods that rely on multiple spatially distinct loci to provide a robust view of the O157:H7 population structure. Each individual multi-locus method in our study pointed to a similar tree structure and the combination of methods in the supernetwork (Figure 3) showed three distinct lineages, two of which were originally proposed by Kim et al. [6] and the third by Zhang et al. [7], adding confidence to the conclusion that these lineages exist.

The results of SNP genotyping over 500 O157:H7 isolates found hyper-virulent clade 8 strains, the causative agents in the spinach- and lettuce-related outbreaks of 2006 in the United States, to be most closely related to sorbitol fermenting *E. coli *O157:NM strains [12]. Based on our *in silico *analyses, these clade 8 strains, which appear to be increasing in prevalence and have a greater association with HUS than other strains [12], are members of lineage I/II (Table 1). Interestingly, *E. coli *O157:H7 strain TW14588 was designated as clade 8 in the initial publication on variation in virulence among the clades [12], but all *in silico *analyses conducted during this study on the whole-genome shotgun sequence available from GenBank (NZ_ABKY) suggests it belongs to clade 2 (lineage I).

It must be recognized that the publicly available genome sequences are not error-free and that this could have affected the architecture of trees that are highly dependent on single nucleotide changes in sequences of target genes, such as SNP-genotyping. However, the *in silico *tree architecture was very similar to that described by Manning et al. [12] based on experimental data; therefore we suspect that this was not a significant source of error in this study.

Differences between lineage I and lineage II strains have been described [29-31], but much less is known about lineage I/II strains with respect to host/disease association or expression of virulence attributes. We have recently demonstrated in a study of *E. coli *O157:H7 strains in Canada that certain phage types (PT)s are specific to O157:H7 lineages [29]. In the latter study, PT2 strains were shown to belong exclusively to lineage I/II, however, others strains in lineage I/II belonged to PTs that were not lineage-restricted e.g. PT23, PT8 and PT1, suggesting that this lineage is widespread and also diverse. Such diversity was also apparent in the examination of the novel regions of O157:H7 DNA, presented in Figure 1.

The Stxs, which are bacteriophage-encoded and the primary virulence factors of *E. coli *O157:H7 [20] show differential distribution among the lineages. The *stx*_2 _gene was found in nearly all O157:H7 lineage I and lineage I/II strains [12,29], while the *stx*_1 _gene was absent in lineage I/II strains but present in nearly all other strains studied. Additionally, Ziebell et al. [29] found the *stx*_2c _gene in 96.7% of lineage II strains, 50.0% of lineage I/II strains and 1.8% of lineage I strains, while Manning et al. [12] found *stx*_2c _to be present in 57.6% of clade 8 strains. This SNP genotyping study also found a significant relationship between the presence of *stx*_2 _in conjunction with *stx*_2c _among strains of clade 8, in that no other clade associated with human illness displayed this combination. This is not surprising as lineage I strains only rarely contain *stx*_2c _despite their high association with human disease and lineage II strains nearly always contain *stx*_2c _despite a rare association with human disease [6]. It is unlikely that the combination of *stx*_2 _and *stx*_2c _alone is the reason for the hyper-virulence of lineage I/II strains, as the presence of *stx*_2c _is nearly ubiquitous among bovine-associated lineage II strains. The SNP genotyping study cited above only considered isolates associated with human disease; therefore it is likely that few lineage II strains were included in the study. A study by Friedrich et al. [32] examining *stx*_2 _subtypes and their association with clinical symptoms found *stx*_2c _to be the only subtype besides *stx*_2 _present in strains isolated from cases of HUS, but found no correlation between the presence of *stx*_2c _and the development of HUS. It has recently been shown that the level of Stx_2 _production is greater in lineage I strains than lineage II strains [33] so it may be that lineage I/II strains implicated in cases of HUS simply produce more toxin than other O157:H7 strains. However, it is possible that other factors possessed by the hyper-virulent lineage I/II group strains are responsible for their greater virulence in humans, and remain to be discovered.

The findings in this study highlight the need for a common genotyping approach, as it is evident that the same groups of genetically related strains have been given multiple designations based on the use of different comparative genotyping methods. This need exists for epidemiological studies of outbreak strains, where strain discrimination is the primary focus, as well as for population genetic studies where genomic information is of central importance. The value of being able to compare a pattern produced in a particular laboratory to one found in a national central database has made PFGE and PulseNet very useful in tracking outbreaks that are widely disseminated [34], despite the fact that PFGE is labour intensive and difficult to standardize [35]. As this common approach in identifying outbreak strains has been useful for a pattern-based method such as PFGE, approaches based on a multi-locus sampling of the genome could take this centralized database concept and extend it to contain presence/absence data for specific loci, SNPs, and other measures of heterogeneity. In this way, whether the goal is identifying an outbreak source or using information for a population-based study, the data would be available in a central repository. This type of system would be important in monitoring and identifying the emergence of new clones of O157:H7, such as the hyper-virulent lineage I/II/clade 8 strains and recognizing other changes in the population when genotyping *E. coli *O157:H7 strains associated with disease outbreaks.

The availability of whole genome sequence information has led to the development of new genotyping methods that are easier to perform than traditional methods, more discriminatory and more informative with respect to genotype and phenotype. It is interesting that the approaches targeting genetic polymorphisms within conserved genes and those targeting genetic changes based on gene insertion/deletion events converge to give a similar picture of *E. coli *O157:H7 strain relationships. Such concordance of methods has been previously demonstrated with mCGH and MLST for *Campylobacter jejuni *[36] and *Streptococcus pneumoniae *[37]. However, given that methods differ greatly in terms of the time required for the analysis, labour and equipment required, need for expertise, freedom from subjectivity in interpretation of the data and portability of the genotyping results from one laboratory to another, there is considerable advantage in selecting a method that is simple, extensible and easily portable. While some typing methods are better than others in these aspects, most of the multi-locus typing methods examined produced a similar tree architecture. This suggests a "common typing language" is possible at least in the context that genotypes derived using different methods can be integrated and communicated in a broader framework. While it was shown that multiple methods converge to provide a similar picture of the O157:H7 population structure, we are not advocating the routine use of multiple genotyping methods but rather the use of methods based on comparative genomics.

With the genomic sequencing revolution well under way, the ability to harvest novel sequence information in a timely fashion from new genomic sequences will become increasingly important and the ability to include and compare *in silico *results to those from traditional laboratory experiments will become necessary.

The results of this study suggest that genotyping approaches based on common comparative genomic data are likely to form the basis for the next-generation of analytical tools used for both population-based comparative genotyping and epidemiological studies.

## Conclusion

A combined analysis of several genotyping methods and supernetwork construction confirm that the *E. coli *O157:H7 population is distributed among three major lineages. Recent work using SNP genotyping has identified an emergent hyper-virulent clade of public health importance among nine SNP-based clades of O157:H7. However, due to the lack of a common genotyping language, this designation has not been relatable to other genotype classification schemes. Using *in silico *analyses of multiple genome-sequenced strains we were able to integrate the overall diversity derived from individual methods into a more complete picture of the O157:H7 population structure and show that based on currently available O157:H7 genome sequence data, lineage I/II and clade 8 strains are synonymous. Further, some factor or factors other than the simple presence of *stx*_2c _are responsible for hyper-virulence among strains of this group. We have also previously shown that all PT2 strains examined belonged to lineage I/II. Future molecular typing and epidemiological studies should be aimed at providing genotyping data based on comparative genomic analyses. These data should be stored centrally and accessed locally in an easily transferable, relatable and extensible format that will form the basis of a common genotyping language.

## Methods

### Genome-sequenced strains used in the analyses

Our analyses utilized the complete and fully annotated genomes of K12 MG1655 and *E. coli *O157:H7 strains EDL933 and Sakai. Additionally, 14 *E. coli *O157:H7 whole-genome shotgun sequences from GenBank were included in the study, along with two whole-genome shotgun sequences that were sequenced in-house: EC71074, an *E. coli *O157:H7 strain and EC33264, an *E. coli *O145:NM strain (Table 1).

### *In silico *lineage typing

The LSPA6 primer sequences [8] were utilized for *in silico *polymerase chain reaction (*is*-PCR) experiments to determine the lineage types of the strains in Table 1. The *is*-PCR method involved BLASTN searches [38] of the primer sequences combined with data processing in Microsoft Excel to determine the expected band size; any primer sequence with less than 80% sequence identity in a BLASTN search was considered absent.

### Shiga-toxin encoding bacteriophage insertion site typing

As described by Besser et al. [9] the primer sequences targeting regions of bacteriophage insertion were subjected to *is*-PCR, using a sequence identity of 80% as a positive threshold. Data were given discrete character scores, with 0 representing absence and 1 representing an amplified band. In cases where bands of more than one size were found for the same primer set, the digits 2 through 5 were used to denote bands of additional size.

### Comparative genomic fingerprinting (CGF)

Twenty-three of the most variable loci among *E. coli *O157:H7 strains were targeted by the comparative genomic fingerprinting method of Laing et al. [13]. The 80 strain binary dataset from the Laing et al. study was used, along with *is*-PCR results from the strains in Table 1, using a sequence identity of 80% as a positive threshold. The *is-*PCR data were converted to binary characters, with 0 for absence and 1 for presence.

### Genomic *in silico *subtractive hybridization (GISSH)

The method for determining the existence of 417 genomic regions larger than 500 bp that are absent from the genomes of EDL933 and Sakai but present in other *E. coli *O157:H7 whole genome shotgun sequences will be described in detail elsewhere (Laing et al. in preparation). Each of the novel regions was split into contiguous segments of 500 bp, creating 1456 segments representing the original set of novel regions.

BLASTN was used to determine the distribution and these 1456 novel regions among the strains in Table 1. A segment containing less than 80% total sequence identity was considered absent and denoted by a 0, while a region containing greater than or equal to 80% total sequence identity was considered present and denoted by a 1.

### Single nucleotide polymorphism (SNP) analysis

Manning et al. [12] recently described the existence of 96 SNPs among *E. coli *O157:H7 strains. The sequence of each SNP-containing locus in *E. coli *O157:H7 strain Sakai was used to conduct BLASTN comparisons of all 96 SNPs in each genomic sequence. Data were scored as single-letter nucleotides for either version of the SNP as put forth by Manning et al. with the following exception: in the Manning et al. study, *E. coli *O157:H7 strain Sakai locus ECs0606 is listed as having a SNP of A117G, whereas our data analysis found the SNP to be A117C.

### Microarray comparative genomic hybridization (mCGH)

Zhang et al. [7] conducted a comprehensive microarray analysis of 31 *E. coli *O157:H7 strains using probe hybridization data for 6057 open reading frames. Analysis of those data found that the 50 mer probes used in the microarray experiments shared sequence identity of at least 80% to one or more of the three control strains (K12 MG1655, EDL933 and Sakai) in 6021 of the 6057 probes. The positive threshold used for *in silico *microarray hybridization was 80% and therefore the 36 ORFs with probes having less than 80% homology in all three control strains were not used. The *in silico *microarray hybridization used BLASTN to test the presence of each ORF in every strain in Table 1. Probes with identity greater than or equal to 80% were scored as present and everything else as divergent. Results were converted to a binary format for subsequent analyses with 1 representing a present ORF and 0 a divergent one.

Zhang et al. used the used the same MWG probe set as Wick et al. [11], which was used to determine the stepwise emergence of O157:H7 strains from an O55:H7-like ancestor. In their study, Wick et al. included two sorbitol-fermenting strains (CB2755 and 493/89) and three O55:H7 strains (Dec 5d, TB182A and 5905) that were absent from the study by Zhang et al. To expand the scope of our study, binary data for these five strains were included, with any loci not included in the Wick et al. study scored as missing and denoted by "?".

### Multi-locus variable number tandem repeat analysis (MLVA)

The MLVA PCR scheme described by Hyattia-Trees et al. [10] was used *in silico *to identify the number of repeats at each locus, which is based on the number of repeats at nine variable loci within the O157:H7 genome. In accordance with the reference, partial repeats were rounded to the nearest whole number to generate the final set of data.

### Construction of dendrograms

The character data for each of the six methods were converted to Nexus format [39] and imported to PAUP* v4.0 [40], where the data were analyzed using maximum parsimony. The tree space was thoroughly sampled using 1000 random sequence additions, with 10 trees kept per search using the following PAUP* command: "hsearch enforce = no start = stepwise addseq = random nreps = 1000 nchuck = 5 chuckscore = 1". The maximum parsimony tree for each set of data was saved and visualized in SplitsTree v4.0, where K12 was used as the outgroup.

### Construction of supernetworks

The six maximum parsimony trees representing the six datasets comprised of the 19 strains in Table 1 were imported to Splitstree v4.0 and a supernetwork was created using Z-closure with 1000 iterations and the un-weighted mean distance of each tree to generate an equal angle representation of the data. A second supernetwork was created in an analogous fashion; however the CGF and microarray trees included data from the previously published studies of Laing et al [13], Wick et al. [11] and Zhang et al. [7].

## Authors' contributions

CRL planned and carried out experimental work, data analysis and writing of the manuscript. CB carried out experimental work. ENT helped with data analysis and with revision of the manuscript. YZ planned experimental work and helped with revision of the manuscript. MAK assisted with project planning and revision of the manuscript. JET helped with project planning and revision of the manuscript. VPJG planned experimental work, contributed to data analysis and with preparation of the manuscript. All authors have read and approved the final manuscript.

## Supplementary Material

Additional file 1 ***In silico *data files**. A Microsoft Excel workbook containing the dataset for each of the six *in silico *typing methods. Click here for file

Additional file 2 **Maximum parsimony trees from the *in silico *analyses**. The maximum parsimony trees created from the *in silico *datasets, which were used in the creation of Figures 2 and 3. Click here for file
