# Supplementary material for: In silico genomic analyses reveal three distinct lineages of Escherichia coli O157:H7, one of which is associated with hyper-virulence
Source: BMC Genomics. 2009 Jun 29;10:287. doi: 10.1186/1471-2164-10-287 (PMC2719669; doi:10.1186/1471-2164-10-287)

Fig. S1- *In silico* Stx-phage integration site typing

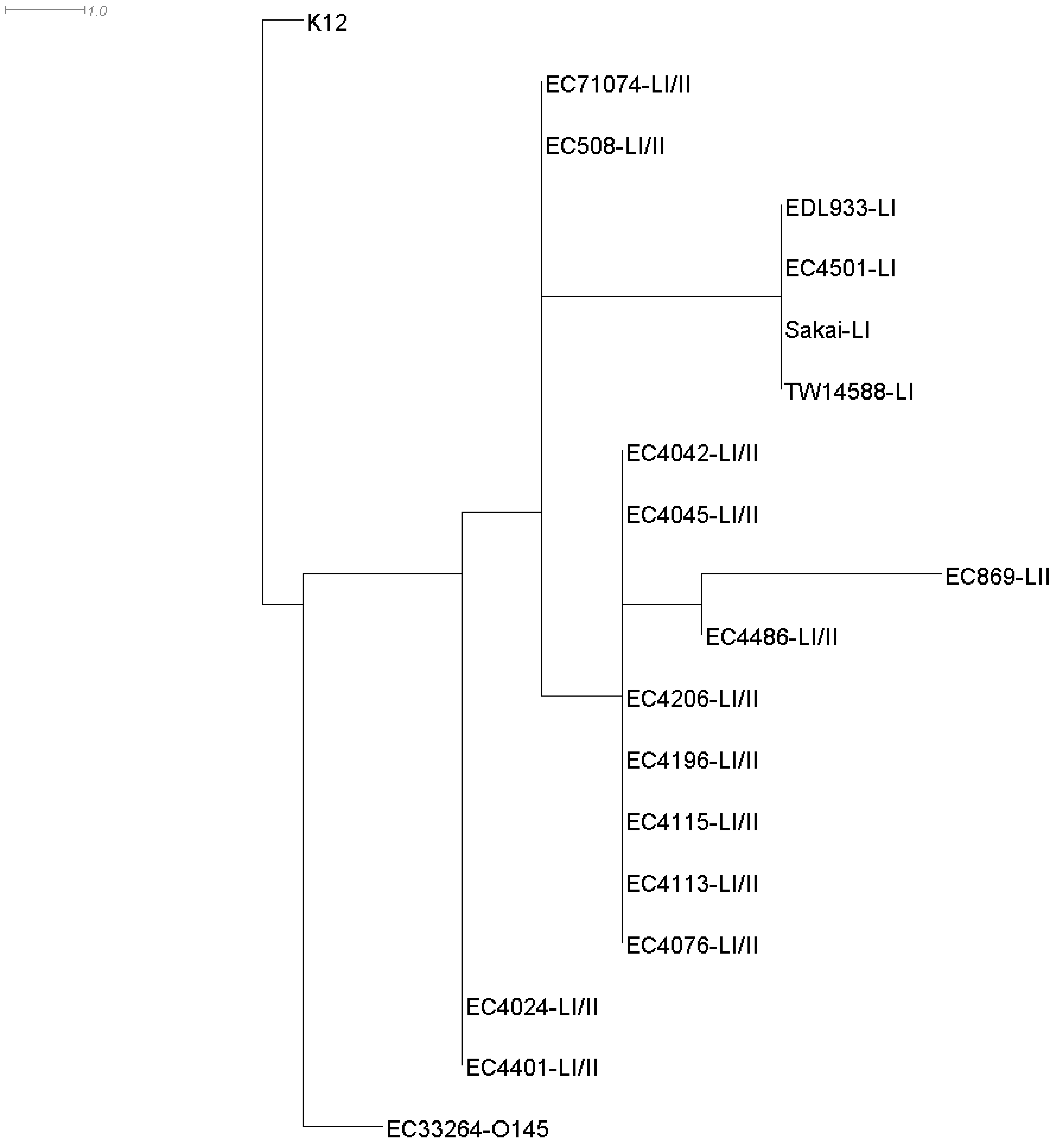

Fig. S2- *In silico* MLVA

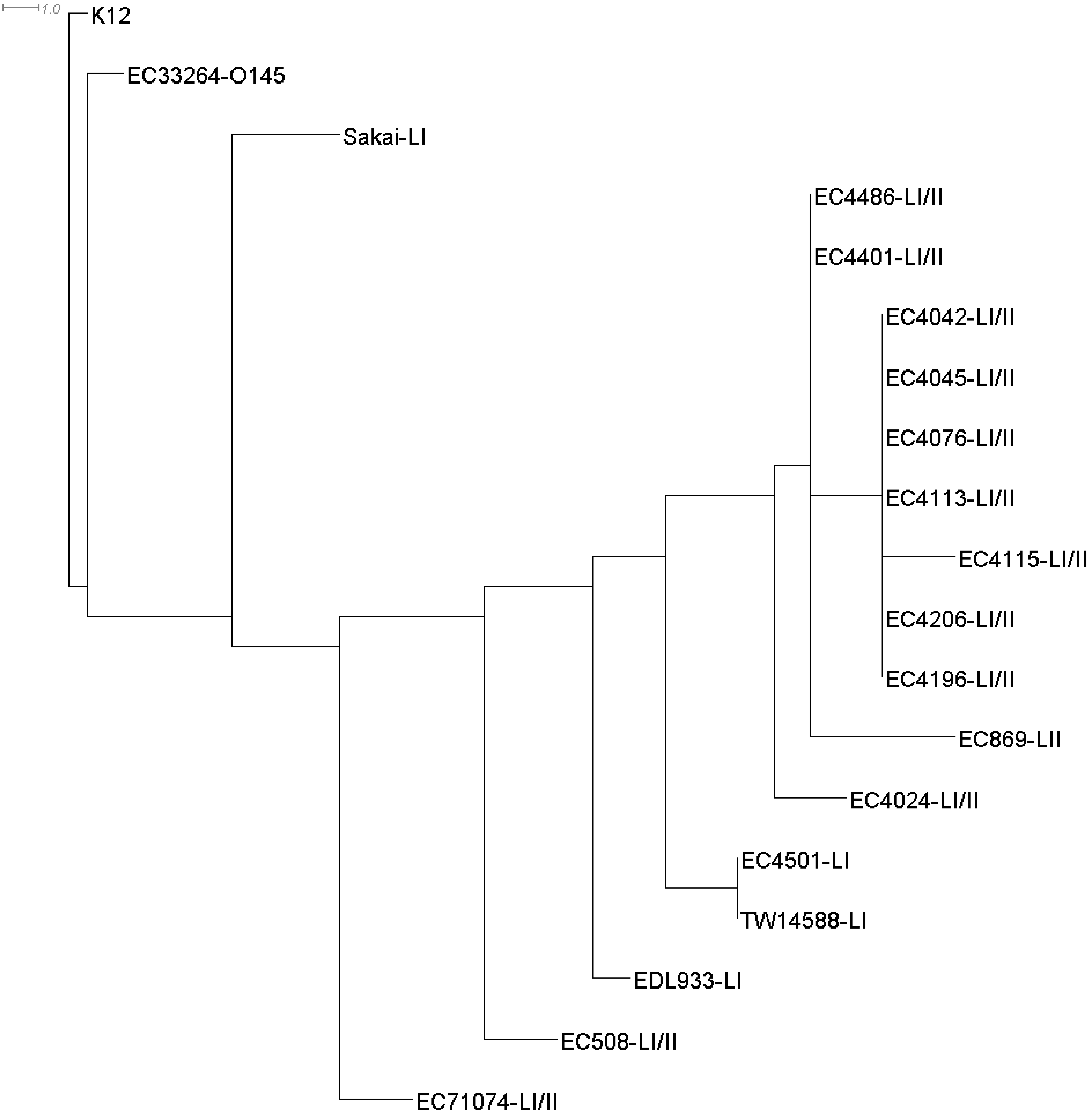

Fig. S3- *In silico* CGF

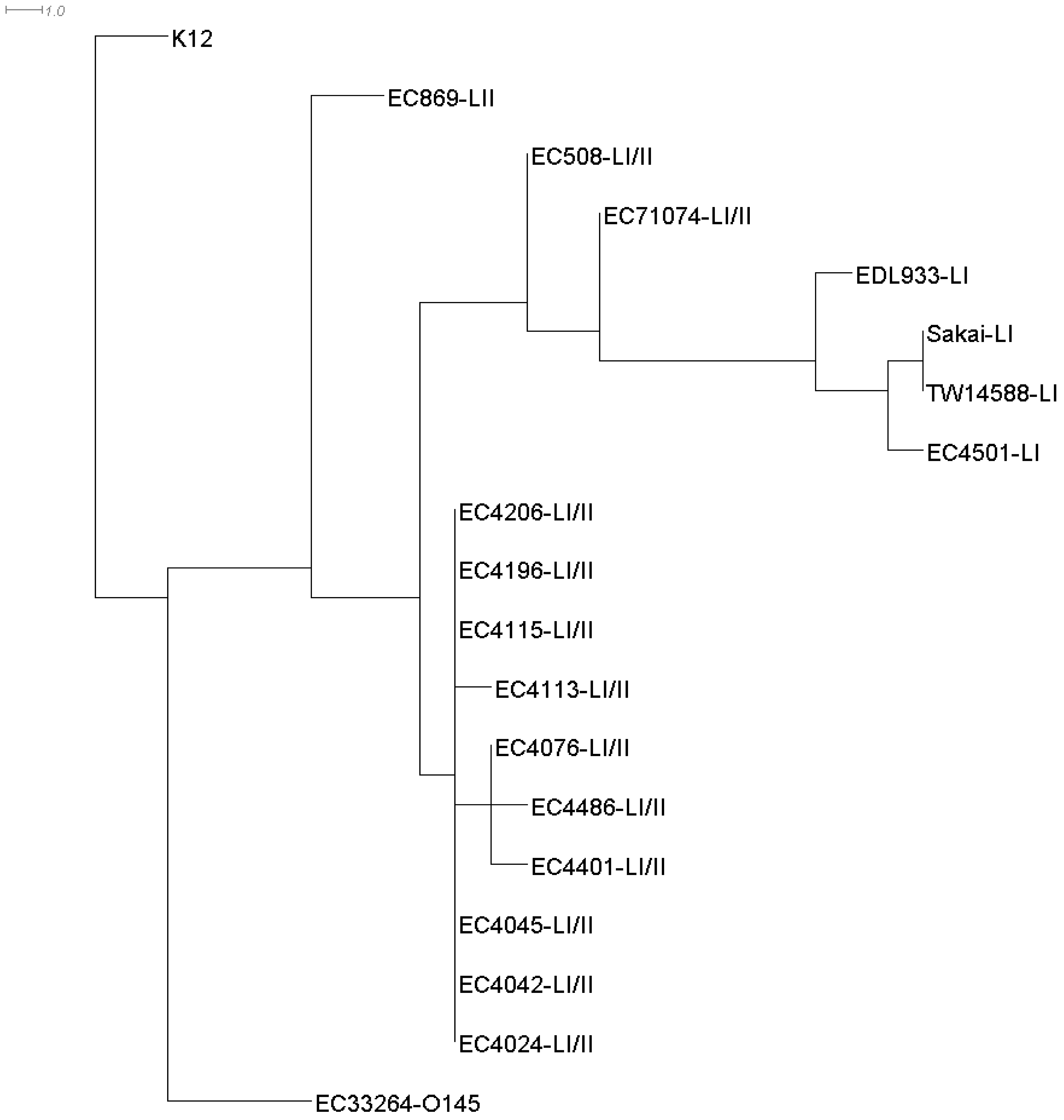

Fig. S4- *In silico* SNP typing

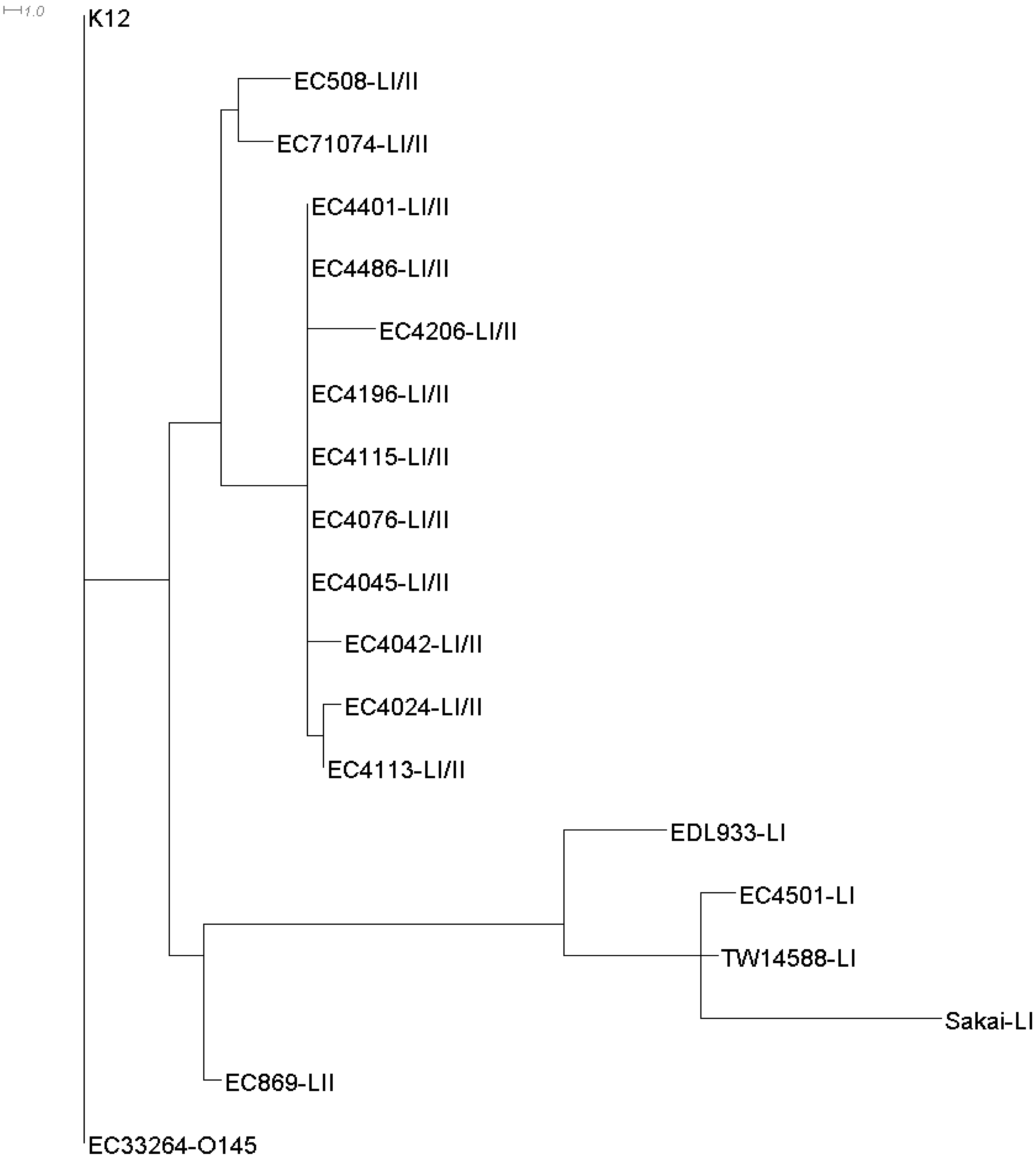

Fig. S5- *In silico* mCGH

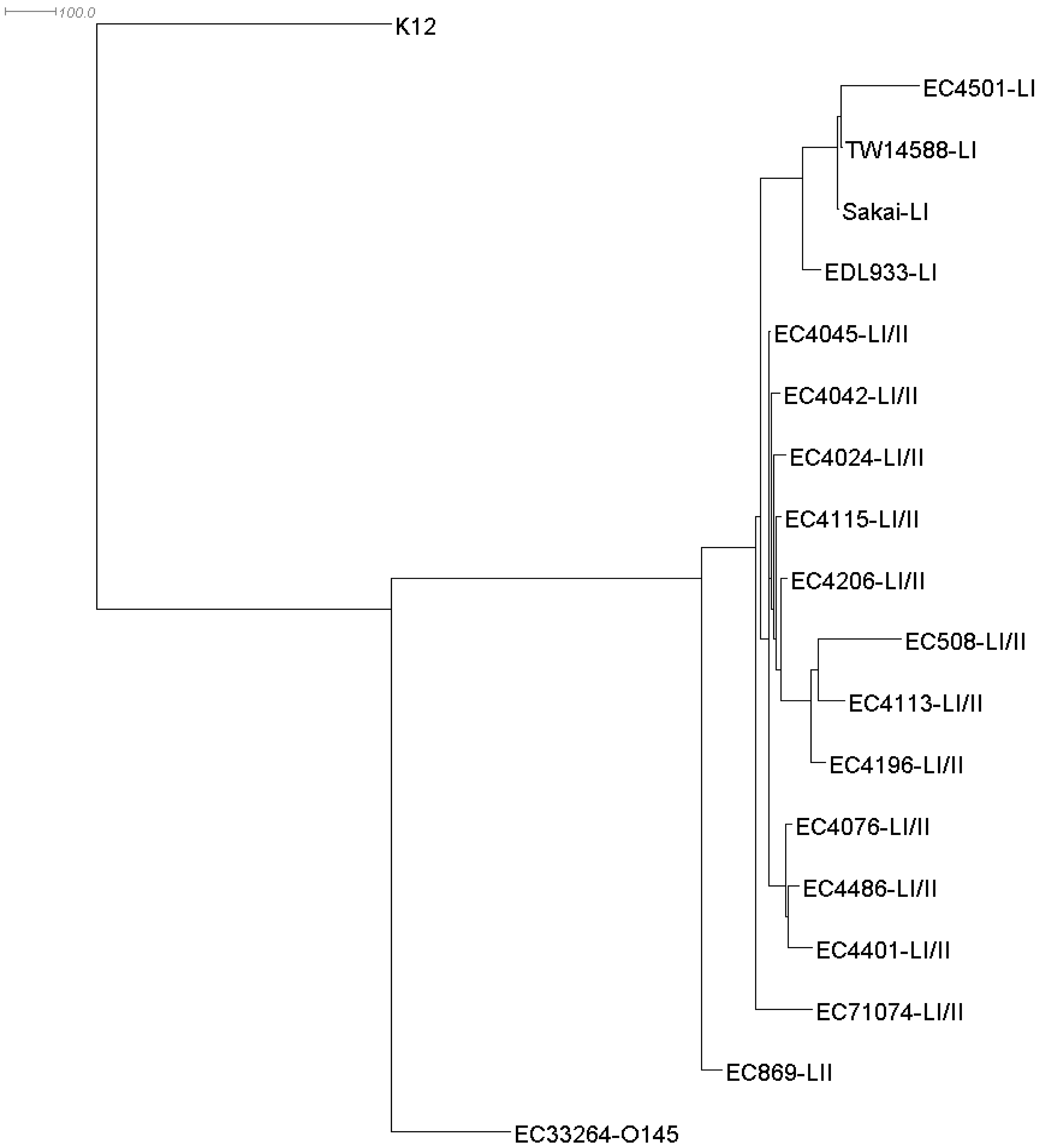

Fig. S6 *In silico* novel region distribution typing

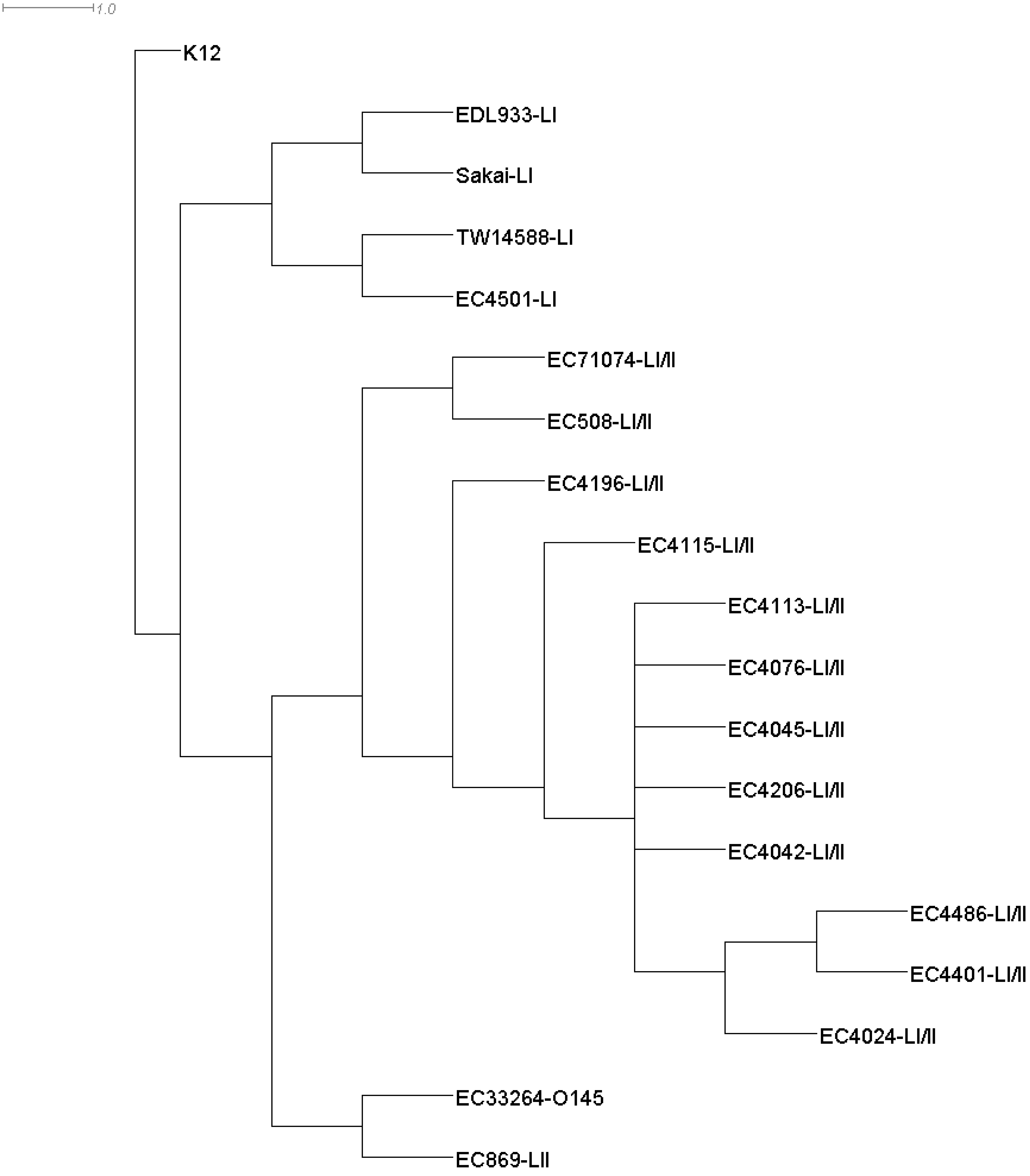

Fig. S7 *In silico* and experimental CGF

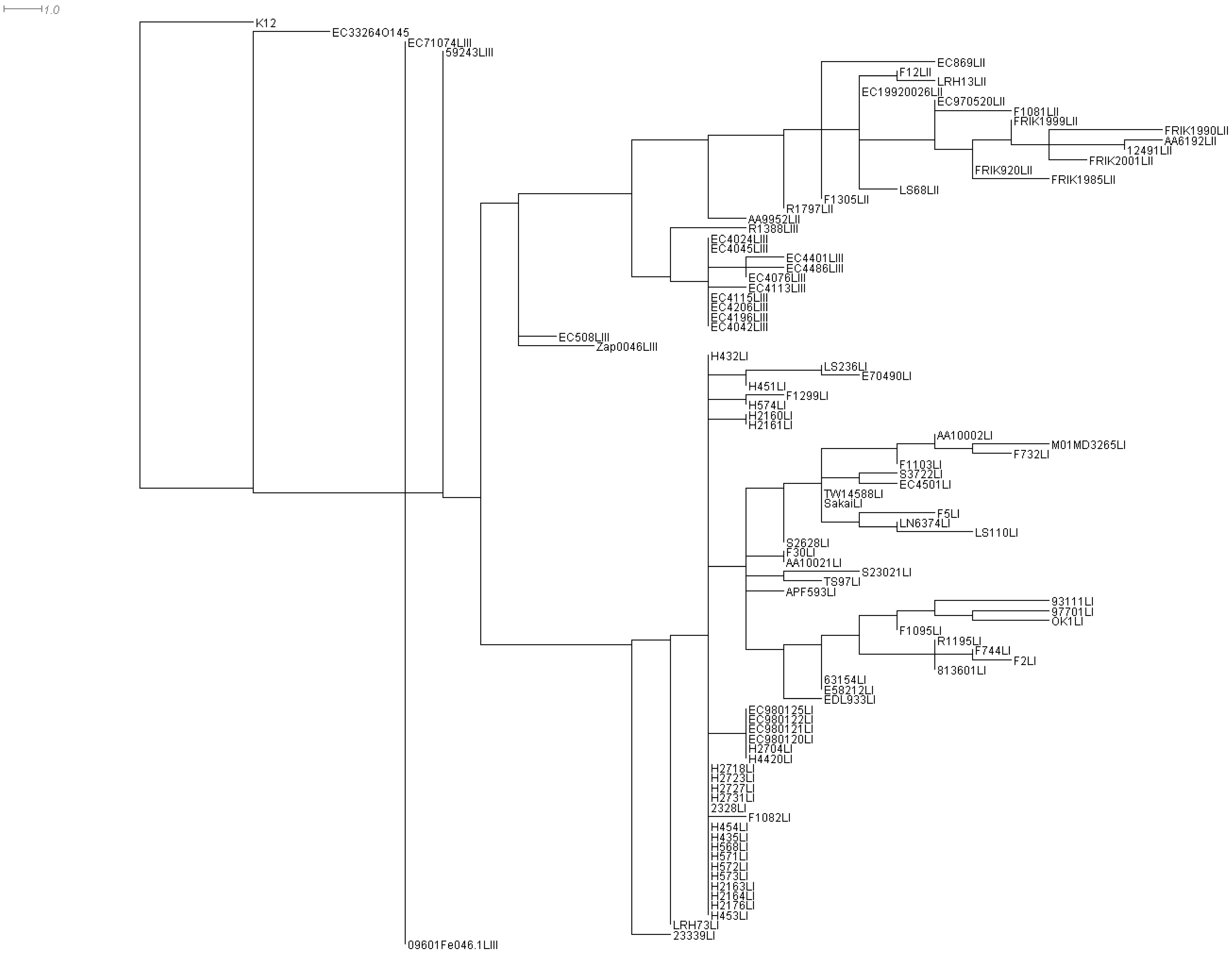

Fig. S8 *In silico* and experimental mCGH

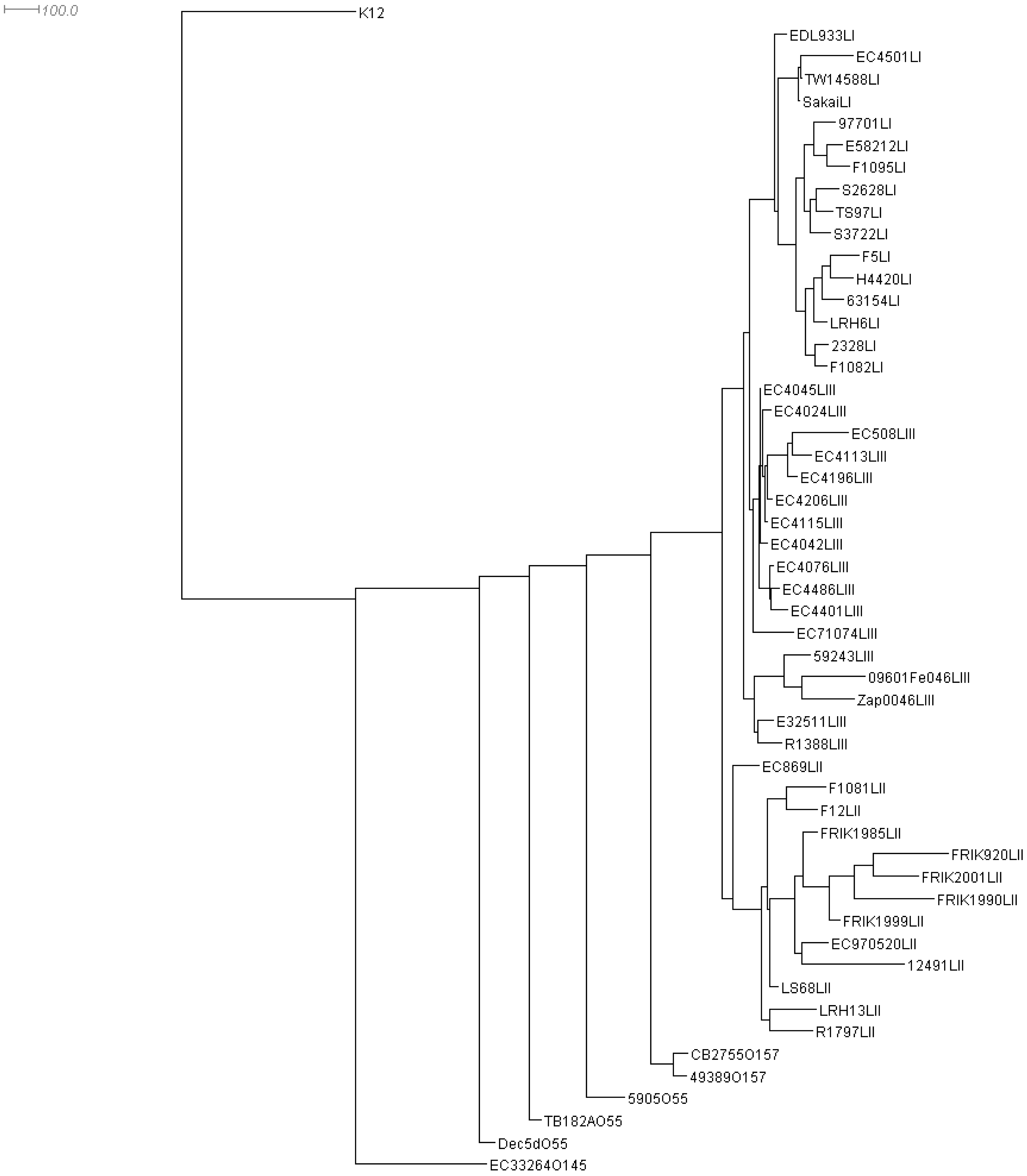

Supplement: Additional file 2 — Maximum parsimony trees from the in silico analyses. The maximum parsimony trees created from the in silico datasets, which were used in the creation of Figures 2 and 3. [file 1471-2164-10-287-S2.pdf]
